# The effect of HIV-1 Vif polymorphisms on A3G anti-viral activity in an in vivo mouse model

**DOI:** 10.1186/s12977-016-0280-y

**Published:** 2016-06-30

**Authors:** Cristhian Cadena, Spyridon Stavrou, Tomaz Manzoni, Shilpa S. Iyer, Frederic Bibollet-Ruche, Weiyu Zhang, Beatrice H. Hahn, Edward P. Browne, Susan R. Ross

**Affiliations:** Department of Microbiology, Perelman School of Medicine, University of Pennsylvania, Philadelphia, PA USA; Department of Microbiology and Immunology, University of Illinois at Chicago College of Medicine, Chicago, IL 60612 USA; Department of Medicine, Perelman School of Medicine, University of Pennsylvania, Philadelphia, PA USA; Koch Institute for Integrative Cancer Research, Massachusetts Institute of Technology, Cambridge, MA USA; Division of Infectious Diseases, Department of Medicine, University of North Carolina, Chapel Hill, NC USA

**Keywords:** APOBEC3, HIV, Vif, Cytidine deaminase, Murine leukemia virus

## Abstract

Humans encode seven *APOBEC3* proteins (A–H), with A3G, 3F and 3H as the major factors restricting HIV-1 replication. HIV-1, however, encodes Vif, which counteracts A3 proteins by chaperoning them to the proteasome where they are degraded. Vif polymorphisms found in HIV-1s isolated from infected patients have varying anti-A3G potency when assayed in vitro, but there are few studies demonstrating this in in vivo models. Here, we created Friend murine leukemia viruses encoding *vif* alleles that were previously shown to differentially neutralize A3G in cell culture or that were originally found in primary HIV-1 isolates. Infection of transgenic mice expressing different levels of human A3G showed that these naturally occurring Vif variants differed in their ability to counteract A3G during in vivo infection, although the effects on viral replication were not identical to those seen in cultured cells. We also found that the polymorphic Vifs that attenuated viral replication reverted to wild type only in A3G transgenic mice. Finally, we found that the level of A3G-mediated deamination was inversely correlated with the level of viral replication. This animal model should be useful for studying the functional significance of naturally occurring *vif* polymorphisms, as well as viral evolution in the presence of A3G.

Apolipoprotein B mRNA editing enzyme, catalytic polypeptide-like (A3) proteins are cytidine deaminases that play important roles in antiviral intrinsic immunity. Humans encode seven genes (*A3A*–*A3H*), and several of these potently restrict HIV-1 by causing G-to-A hypermutation of the viral genome as well as by deaminase-independent mechanisms [1]. HIV and most other lentiviruses, however, contain Viral Infectivity Factor (*vif*) genes, which encode proteins that counteract the antiviral activity of A3 proteins [2–4]. In cells producing *vif*-deficient HIV-1, A3 proteins are packaged into progeny virions via interaction with the nucleocapsid (NC) protein and viral RNA. However, in cells producing wildtype virus, Vif binds A3D, A3F, A3G and certain A3H haplotypes. Although efficiencies vary, Vif binding targets these proteins for ubiquitinylation and degradation in the proteasome through interactions with a number of host cellular factors, including CBF-β, Cul5 and elongins, thereby preventing their packaging and overcoming the anti-viral activity [5–12]. In vitro studies have shown that naturally occurring polymorphisms, in many cases resulting in single amino acid residue changes, enhance or attenuate the ability of Vif to interact with and antagonize A3G as well as other A3s. Furthermore, it has been shown that the anti-A3G activities of Vifs found in HIV-1s isolated from Elite Controllers are significantly lower than those derived from non-controllers, suggesting that this host anti-viral activity is important for the effective control of infection in vivo [13]. It has also been suggested that partial neutralization of A3G may increase HIV-1 sequence diversity and promote the emergence of immune escape variants as well as variants with altered co-receptor usage [9, 14–16]. Due to the potential role of A3G in the diversification of HIV-1, as well as the interest in the development of inhibitors that target the A3G–Vif interaction, it is important to characterize the impact of *vif* polymorphisms on A3G-mediated virus restriction in vivo.

Our lab pioneered the study of A3 genes in vivo using transgenic mice expressing individual human A3 proteins [17]. Two A3G transgenic mice strains were developed on a mouse A3 knockout (KO) background, expressing high and low levels of the transgene in the absence of a mouse A3 ortholog; importantly, the level of A3G expression in the A3G^high^ strain was similar to that seen in human peripheral blood mononuclear cells, while the levels in the A3G^low^ strain were approximately 30-fold lower [17]. Our previous work demonstrated potent inhibition of retrovirus replication by A3G in these mice and showed that the level of virus replication was proportional to the level of A3G expression and cytidine deamination of the viral genomes. Furthermore, we showed that A3G-mediated restriction could be overcome in vivo by a Friend murine leukemia virus (F-MLV) encoding the HIV-1 NL4-3 *vif* gene, derived from a lab-adapted HIV-1 molecular clone; expression of Vif led to degradation of A3G in splenic extracts, decreased packaging into virions and increased cytidine deamination of viral genomes [17]. The in vivo cellular targets of MLV infection are similar to HIV-1, namely sentinel cells, such as macrophages and dendritic cells, and lymphocytes [18–20].

Here, we tested whether our A3G transgenic mouse models could be used to assess the effect of naturally occurring *vif* polymorphisms on the ability of this accessory protein to counteract host A3G restriction [21, 22]. For this, we first developed five F-MLV-vif variant viruses that encoded *vif* alleles that differ only at a single amino acid position from the NL4-3 HIV-1 molecular clone; these polymorphisms were previously identified in HIV-1 strains identified in patient PBMCs (Fig. 1a) [21, 22]. The amino acid residue changes include three found in non-progressors (W11R, Y40H and G143R), one that arose after infection of non-progressor PBMCs with patient-derived HIV-1s (K22E) and one found as a common mutation in patients who failed highly active anti-retroviral therapy (K22H). Previous cell culture studies revealed that Y40H, K22E and K22H had reduced ability to counteract A3G, while W11R and G143R were as active as the NL4-3 Vif. To introduce these variant amino acids into F-MLV, the NL4-3 vif sequence was cloned into pcDNA3.1/myc-his (Invitrogen) and the specific amino acid changes were introduced by site-directed mutagenesis (QuikChange II XL Site-Directed Mutagenesis Kit, Agilent). The mutated *vif* genes were then inserted into a plasmid containing a replication-competent F-MLV-2A molecular clone, as previously described [23] (Fig. 1a). Virus stocks were produced by transfection of 293T cells and supernatants were collected 48 h post-transfection. Amplification of viruses to generate high-titer stocks was performed as described [23]. Viral RNA from these stocks was PCR-amplified to ensure that the *vif* gene was retained during passage; amplification of the same RNAs with *env* primers served as controls (Fig. 1b) and the *vif* regions were sequenced to ensure that the targeted mutations were retained (not shown). Additionally, we examined Vif levels after transfection of the viral constructs into 293T virus producer cells by western blot and showed that each of the mutants was expressed at similar levels in virus-producer cells (Fig. 1c).

**Fig. 1:**
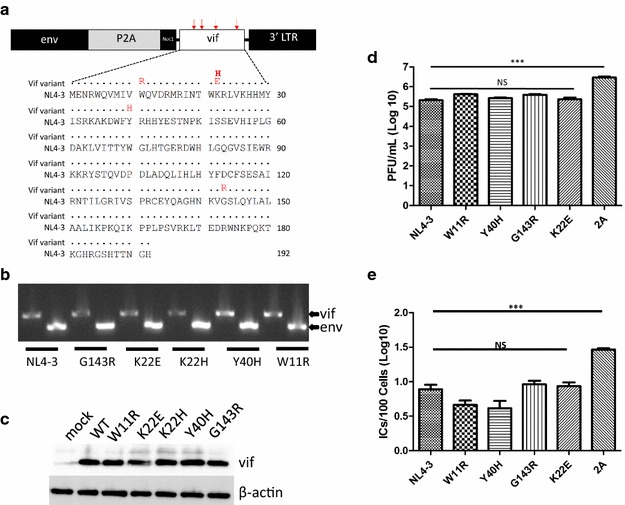
**a** *Top* a plasmid encoding the full‐length replication‐competent clone Friend MLV was modified by adding a 2A peptide sequence (P2A) from picornaviruses, in frame with the C terminus of the envelope gene, followed by a Not1 restriction site and a stop codon. The *vif* gene was cloned in frame into the Not1 site to generate F-MLV‐2A‐vif. *Bottom* the Vif protein sequence of the NL4-3 molecular clone is shown. The single amino acid residue changes used for this study are highlighted in red at positions a11, a22, a40, and a143. **b** PCR of RNA isolated from viruses propagated in vitro using *vif*- and *env*-specific primers. **c** Western blot analysis of cell extracts from virus-producer cells. 293T cells were co-transfected with the indicated F-MLV-vif virus and 24 h post-transfection, equal amounts of cell lysates were analyzed by Western blot, using anti-Vif and anti-β-actin antisera. **d** Replication of F-MLV-Vif variants in vitro. *Mus dunni* cells were infected at an MOI = 0.1. Supernatants were collected 6 days post-infection and viral titers were measured by plaque assay. The data represent the average of 3 experiments, with the *error bars* showing standard deviations. **e** 2 day-old A3 KO mice were infected with 10^4^ PFU of each virus intraperitoneally. Splenocytes were isolated at 16 dpi and tenfold dilutions of these cells were co-cultured with *Mus dunni* cells for 3 days. Viral titers were assessed by plaque assay. The data represent the average of at least 5 mice per group, with the *error bars* showing standard deviations. ***P ≤ 0.001; *NS* not significant (one-way ANOVA)

As the replication efficiency of the F-MLV variant viruses could differ due to the insertion of *vif* or the introduction of specific mutations, we first assessed the replication kinetics of these viruses in culture over a 9-day period. All *vif*-containing viruses replicated with similar kinetics (not shown), with peak viremia observed at 6 days post-infection (dpi) (Fig. 1d). Wildtype F-MLV (F-MLV-2A) grew to significantly higher titers at 6 dpi, indicating that insertion of the HIV-1 *vif* gene attenuated viral replication. However, the introduction of single amino acid residue changes into the NL4-3 vif in F-MLV did not significantly affect the replication of any of the F-MLV-vif variants (Fig. 1d). Similar results were obtained when the viruses were inoculated into A3 KO mice (Fig. 1e).

Next, we compared infection of A3 KO and both human A3G transgenic mouse lines with the different F-MLV-Vif variants as well as F-MLV-2A. Because the range of basal expression of A3G at the RNA level in PBMCs from HIV-1 uninfected individuals has been reported to be >tenfold [24], we tested the efficacy of each of the variant Vifs in both A3G^high^ and A3G^low^ mice, thereby mimicking the variation in A3G expression seen in humans. Two-day old pups of each strain were injected intraperitoneally with 10^4^ PFU of each virus. Spleens were collected at 16 dpi and infection levels were determined by infectious center (IC) assays using a focal immunofluorescence assay, as previously described [17].

As shown previously, A3G restricted F-MLV-2A proportionally to its level of expression, with infection reduced by half a log and 1 log in A3G^low^ and A3G^high^ mice, respectively, relative to infection in KO mice. By contrast, as we showed previously, F-MLV-Vif (NL4-3) replication was inhibited in the A3G^high^ strain by only half a log, while no differences were observed between A3G^low^ and KO mice, suggesting that the NL4-3 Vif fully compensated low levels of A3G-mediated restriction (Fig. 2a) [25]. F-MLV-G143R behaved similarly to NL4-3; this was also seen in the in vitro studies [21]. However, several other mutants examined in vivo showed different results than in vitro. The K22E and K22H mutations which were previously shown to weakly counteract A3G’s anti-Vif activity, had no anti-viral activity in either the A3G^low^ or A3G^high^ mice in vivo, producing infection levels similar to the *vif*-virus F-MLV-2A (Fig. 2a) [21, 22]. In fact, K22E was even more attenuated in vivo than F-MLV-2A in the A3G^low^ mice, but this may reflect a reduced ability to replicate (Fig. 1e). Y40H, which appeared to encode a weaker Vif than NL4-3 Vif in vitro, instead was able to counteract A3G in vivo similar to NL4-3 Vif, although there was somewhat greater variability in infection levels in individual mice. Mutant W11R did not show any inhibition by A3G, even in A3G^high^ mice, although in vitro it behaved similarly to NL4-3 Vif. This suggests that the W11R mutation might confer Vif with a more potent A3G neutralization ability than the NL4-3 allele.

**Fig. 2:**
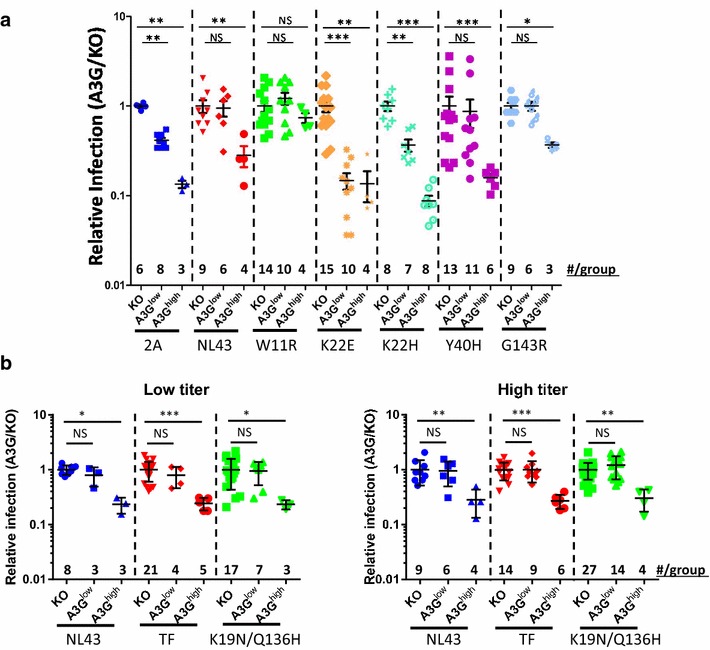
Relative virus titers in the spleens of A3G transgenic mice after infection of with F-MLV-Vif variants. **a** Newborn A3 knock-out or A3G transgenic mice were infected intraperitoneally with 10^4^ PFU of the different F-MLV-Vif variants. Spleens were collected 16 days post infection and splenocytes were isolated for assessment of viral levels by infectivity assays on *Mus dunni* fibroblasts. **b** Newborn mice were also infected with 10^3^ (*left*) or 10^4^ (*right*) PFU of F-MLV-Vif^TF^ or F-MLV-Vif^K19N/Q136H^. Each *point* represents the titer obtained from each individual mouse normalized to the average titer measured in the spleens of A3 KO mice infected with the same virus (A3G/KO). Each *point* represents the viral load measured from an individual mouse; the mean (*horizontal bar*) and standard deviation for each group is shown. The transgenic mice were derived from 2 to 4 litters each; the knockout mice are the littermates of the transgenic mice. *P ≤ 0.05; **P ≤ 0.01; ***P ≤ 0.001; *NS* not significant (Mann–Whitney *T* test)

Since the NL4-3 *vif* counteracted A3G in the context of F-MLV, we used this transgenic mouse model to examine the *vif* genes of a pair of matched transmitted founder (TF) and 6 months (6-mo) consensus infectious molecular clones (IMC) from a longitudinally studied subject (CH470), which differed in their sensitivity to type 1 interferons [26]. Since these two IMCs encoded Vif proteins that differed in 3 amino acids, we hypothesized that two non-conservative changes (K19N/Q136H) might be responsible for altered A3G counteraction. To test this, we cloned the TF and K19N/Q136H mutant *vif* alleles into the F-MLV-2A backbone and examined their function in vivo. Similar to what was seen for F-MLV-NL4-3, mice bearing the CH470-derived *vif*s replicated less efficiently than the *vif*-parent (not shown). Two different virus inocula were used to infect the different mice, 10^3^ and 10^4^ PFU/mouse. Both CH470 *vif* containing viruses replicated to levels similar to that seen with the virus containing NL4-3 Vif after inoculation with either virus amount, fully counteracting A3G in mice with low level expression and partially restoring infectivity in mice with high level expression (Fig. 2b). No difference was seen between the TF and K19N/Q136H mutant *vif*-containing viruses, indicating that these particular amino acid changes did not alter Vif/A3G interactions. Thus, the observed differences in IFN sensitivity of the CH470 TF and 6-mo IMCs are unlikely to be explained by differential A3G counteraction.

To ensure that the viruses propagated in vivo retained *vif*, we PCR-amplified DNA isolated from infected splenocytes and showed that all of the virus populations had *vif*-containing viruses (Fig. 3a). We also cloned and sequenced the *vif*s from the different viruses in A3KO, A3G^high^ and A3G^low^ mice (Table 2). Interestingly, we found that the two most attenuated viruses in the presence of A3G, K22E and K22H, showed reversion to wild type *vif* in both the A3G^low^ and A3G^high^ strains, while for Y40H, which was attenuated only in A3G^high^ mice, we found revertants in this but not the A3G^low^ strain (Fig. 3b; Table 2). No reversion was seen with the fully infectious W11R or G143R viruses.

**Fig. 3:**
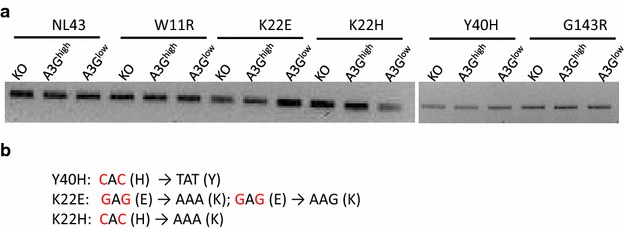
*Vif* sequences are retained in viruses passaged in vivo. PCR analysis. **a** DNA isolated from the spleens of the mice infected with the different viruses was PCR-amplified with primers flanking *vif* (5′-atggaaaacagatggcaggt-3′/5′-ctagtgtccattcattgta-3′). **b** Sequences of the revertant viruses found in the Y20H-, K22E- and K22H-infected mice

Sequencing of F-MLV *env* as well as the various *vif*s after infection showed that, as described in previous in vitro studies and patient samples, the level of restriction by A3G was proportional to the level of G-to-A hypermutation in the virus (Tables 1, 2). As we reported previously, the majority of the G-to-A changes were in the GG motif (not shown) [17]. NL4-3, G143R, W11R, all potent inhibitors of A3G, showed less deamination overall in both mouse strains, while 2A, K22E and K22H, showed more deamination and introduced more stop codons than the other viruses, corresponding to the absence or lack of Vif activity. Y40H, which showed an intermediate phenotype in vitro, also had an intermediate level of deamination (Tables 1, 2).

**Table 1:** Mutation frequencies and reversion analysis of *vif* of F-MLV-Vif proviruses bearing different Vif variants in splenic DNA of infected APOBEC3 KO, A3G^high^ and A3G^low^ transgenic mice

	NL43			W11R			K22E			K22H			Y40H			G143R		
	KO	A3G^Low^	A3G^High^	KO	A3G^Low^	A3G^High^	KO	A3G^Low^	A3G^High^	KO	A3G^Low^	A3G^High^	KO	A3G^Low^	A3G^High^	KO	A3G^Low^	A3G^High^
#G to A	0	2	4	2	1	6	1	14	19	1	10	22	0	5	12	2	2	3
#other	8	7	10	9	8	9	9	9	12	7	9	11	8	10	10	9	8	9
#clones	15	14	14	15	14	15	15	12	15	13	14	14	14	14	14	15	15	14
#bp	8700	8120	8120	8700	8120	8700	8700	6960	8700	7540	8120	8120	8120	8120	8120	8700	8700	8120
G to A freq. ×10^−3^	0.00	0.25	0.49	0.23	0.12	0.69	0.11	2.01	2.18	0.13	1.23	2.71	0.00	0.62	1.48	0.23	0.23	0.37
Other freq. ×10^−3^	0.92	0.86	1.23	1.03	0.99	1.03	1.03	1.29	1.38	0.93	1.11	1.35	0.99	1.23	1.23	1.03	0.92	1.11
#stop codons	0	0	1	0	1	3	1	4	3	2	5	8	0	1	3	1	1	3
#revertants	NA	NA	NA	0/15	0/14	0/15	0/15	1/12	3/15	0/13	1/14	2/14	0/14	0/14	2/14	0/15	0/15	0/15

Analysis was performed on DNA cloned from a single infected mouse in Fig. 2a. F-MLV has a total of 38 GG motifs in the 580 region that was sequenced

**Table 2:** Mutation frequencies in *env* of F-MLV-Vif proviruses bearing different Vif variants in splenic DNA of infected APOBEC3 KO, A3G^high^ and A3G^low^ transgenic mice

	FMLV-2A			NL43			W11R			K22E		
	KO	A3G^Low^	A3G^High^	KO	A3G^Low^	A3G^High^	KO	A3G^Low^	A3G^High^	KO	A3G^Low^	A3G^High^
#G to A	7	27	116	1	2	12	1	1	18	5	57	63
#other	37	19	43	67	28	71	13	6	12	26	34	49
#mice	3	3	3	3	3	4	3	4	3	3	3	3
#clones	42	40	41	42	41	53	49	42	45	50	45	48
#bp	24,612	23,440	24,026	24,612	24,026	31,058	28,714	22,268	26,370	29,300	26,370	28,128
G to A freq. ×10^−3^	0.28	1.15	4.83	0.04	0.08	0.39	0.03	0.04	0.68	0.17	2.16	2.24
Other freq. ×10^−3^	1.50	0.81	1.79	2.72	1.17	2.29	0.45	0.27	0.46	0.89	1.29	1.74
#stop codons	2	4	46	0	1	4	0	0	7	0	15	31

	K22H			Y40H			G143R		
	KO	A3G^Low^	A3G^High^	KO	A3G^Low^	A3G^High^	KO	A3G^Low^	A3G^High^
#G to A	3	29	62	0	10	27	2	7	9
#other	22	28	49	18	21	5	11	13	10
#mice	3	3	3	3	4	3	4	3	3
#clones	44	45	46	45	52	41	41	41	39
#bp	25,784	26,370	26,956	26,370	30,472	24,026	24,026	24,026	22,854
G to A freq. ×10^−3^	0.16	1.10	2.30	0.00	0.33	1.12	0.08	0.29	0.39
Other freq. ×10^−3^	0.85	1.06	1.82	0.68	0.69	0.21	0.46	0.54	0.44
#stop codons	0	14	25	0	0	7	1	1	1

Analysis was performed on DNA cloned from a subset of the infected mice in Fig. 2 (3–4 mice/group; 10–15 sequences/mouse). F-MLV has a total of 58 GG motifs in the 586 bp region that was sequenced

In our panel of F-MLV-Vif polymorphisms, we found viruses that display a range of anti-A3G activity. Infection of transgenic mice with F-MLV encoding the NL4-3 vif and 2A agrees with our previous results, with both A3G strains restricting 2A, but only A3G^high^ restricting NL4-3. In contrast to our previous study where F-MLV-2A infection in A3G^low^ mice was decreased by more than 1 log, here we saw only about a fivefold difference in infection compared to the A3 KO mice. This is likely due to the higher dose of virus used (10 times higher than our previous experiments). Interestingly, W11R Vif showed a stronger phenotype than NL4-3 Vif, as the virus was able to overcome A3G even in the A3G^high^ mice (Fig. 2), although the G-to-A deamination frequency was similar (Table 1). This is in contrast to previous studies in tissue culture, where W11R infection was identical to that seen with NL4-3. K22E, however, showed a weaker phenotype than was seen previously, being restricted by A3G even at low expression levels and exhibiting high levels of cytidine deamination. These differences may be due to the level of A3G expression or to the cell type in which A3G is expressed; the cellular targets of infection in the transgenic mice are likely similar to the natural targets of HIV infection in humans (e.g. sentinel and lymphoid cells).

Humanized mice infected with HIV-1 have also been used to study A3–Vif interactions in vivo [27, 28]. Similar to these studies, which showed that HIV proviral DNA is deaminated, even in viruses containing *vif*, we found that Vif in F-MLV decreased A3G deamination in proportion to transgene expression (Tables 1, 2). Sub-lethal hypermutation by A3 proteins has been suggested to play a role in the diversification of the HIV-1 genome. Polymorphisms in Vif, readily identifiable in HIV-infected patients, have been shown to partially neutralize A3G in vitro, and this might provide the virus with a novel mechanism for diversification. Our transgenic mice represent a unique model system to test these hypotheses, as well as an in vivo model for assessing the efficacy of anti-viral drugs that target A3G–Vif interactions.

There are other A3 proteins, such as A3F, that also are counteracted by Vif and which have been implicated in restricting HIV-1 infection in humanized mice [28]. The construction of A3F- and other human A3-expressing transgenic mice would also permit the testing of drugs that target specific A3–Vif interactions. One advantage of the transgenic mice is that individual A3 proteins can be studied in isolation compared to humanized mice and therefore the interaction of different Vifs with individual A3 proteins can be readily distinguished both in the presence and absence of drugs that counteract these interactions. Since we also showed previously that Vif expressed in the context of MLV leads to APOBEC3G degradation in the transgenic mice, these mice might be useful for testing compounds that target Vif-EloC/B interactions [17].

We also found reversion of F-MLV-Vif viruses which were highly attenuated for infection only in the A3G-expressing mice. Although we only allowed infection to proceed in these experiments for 16 days, we showed previously that MLV infection can be followed in vivo for 6 weeks post-infection [17]. Indeed, we have found that the majority of the A3G^low^ transgenic mice maintain low virus loads for at least 7 months post-infection and do not succumb to leukemogenesis (not shown); it is likely that the A3G^high^ mice will be even more resistant to virus-induced disease. Thus, these mice are likely to be useful to long-term studies of viral escape from host anti-viral factors, as well as virus diversification.

In summary, using novel human A3G transgenic mouse models that express varying levels of A3G as is seen in humans, this study clearly demonstrates that polymorphic *vif* alleles can have differential anti-A3G activity in vivo.
